# Safety and Effectiveness of Monochloramine Treatment for Disinfecting Hospital Water Networks

**DOI:** 10.3390/ijerph17176116

**Published:** 2020-08-22

**Authors:** Isabella Marchesi, Stefania Paduano, Giuseppina Frezza, Luca Sircana, Elena Vecchi, Pietro Zuccarello, Gea Oliveri Conti, Margherita Ferrante, Paola Borella, Annalisa Bargellini

**Affiliations:** 1 Department of Biomedical, Metabolic and Neural Sciences, Section of Public Health, University of Modena and Reggio Emilia, Via Campi 287, 41125 Modena, Italy; stefania.paduano@unimore.it (S.P.); giuseppina.frezza@unimore.it (G.F.); paola.borella@unimore.it (P.B.); annalisa.bargellini@unimore.it (A.B.); 2 University Hospital Policlinico of Modena, Largo del Pozzo 71, 41124 Modena, Italy; sircana.luca@aou.mo.it (L.S.); vecchi.elena@aou.mo.it (E.V.); 3 Environmental and Food Hygiene Laboratory (LIAA), Department of Medical and Surgical Sciences and Advanced Technologies “G.F. Ingrassia”, University of Catania, 95123 Catania, Italy; pietro.zuccarello@unict.it (P.Z.); olivericonti@unict.it (G.O.C.); marfer@unict.it (M.F.)

**Keywords:** monochloramine, disinfection by-products, N-nitrosamines, hospital setting, water networks, *Legionella*

## Abstract

The formation of potentially carcinogenic N-nitrosamines, associated with monochloramine, requires further research due to the growing interest in using this biocide for the secondary disinfection of water in public and private buildings. The aim of our study was to evaluate the possible formation of N-nitrosamines and other toxic disinfection by-products (DBPs) in hospital hot water networks treated with monochloramine. The effectiveness of this biocide in controlling *Legionella* spp. contamination was also verified. For this purpose, four different monochloramine-treated networks, in terms of the duration of treatment and method of biocide injection, were investigated. Untreated hot water, municipal cold water and, limited to N-nitrosamines analysis, hot water treated with chlorine dioxide were analyzed for comparison. *Legionella* spp. contamination was successfully controlled without any formation of N-nitrosamines. No nitrification or formation of the regulated DBPs, such as chlorites and trihalomethanes, occurred in monochloramine-treated water networks. However, a stable formulation of hypochlorite, its frequent replacement with a fresh product, and the routine monitoring of free ammonia are recommended to ensure a proper disinfection. Our study confirms that monochloramine may be proposed as an effective and safe strategy for the continuous disinfection of building plumbing systems, preventing vulnerable individuals from being exposed to legionellae and dangerous DBPs.

## 1. Introduction

The control of *Legionella* contamination in complex water systems is still a critical issue, especially in healthcare settings where *Legionella* colonization of hot water systems is the primary risk factor for Legionnaires’ disease [1]. National and international guidelines, aimed at preventing *Legionella* infections, recommend using biocides for contaminated hot water treatment [2,3]. To date, several physical and chemical disinfection methods have been proposed, but the most effective procedure has yet to be defined [4]. Chlorine-based disinfectants are cheap and widely used but they produce disinfection-by products (DBPs), which can pose potential health risks [5]. Exposure to DBPs is associated with an increased risk of cancer in humans and animals, and concerns regarding human reproduction and development [6,7]. Alternatives to chlorine such as chlorine dioxide, chloramines, ozone, and UV disinfection can be used. Chlorine and each of these disinfectants have different advantages and disadvantages in terms of cost, efficacy and stability, ease of application, pipe corrosion, and types of DBPs [8,9]. The DBPs of most concern include trihalomethanes (THMs) and haloacetic acids formed with chlorine, bromate formed during ozonation, and chlorite typically formed from chlorine dioxide treatment.

Monochloramine is a biocide that has long been used in the USA for the primary disinfection of municipal water supplies [10]. Some studies suggest that the introduction of monochloramine into a municipal water system represents the only community-based intervention that is capable of reducing the incidence of Legionnaire’s disease associated with potable water [11,12]. Other studies report a lower prevalence of *Legionella* colonization in urban water supply systems, using monochloramine instead of free chlorine for disinfecting municipal drinking water [13,14]. On this basis, monochloramine has been used successfully for the secondary disinfection of hospital water networks [15,16,17,18] because it is less aggressive than chlorine and is more persistent in distribution systems. Chloramines produce lower DBP levels than chlorine [19], even if nitrogen by-products have been detected in chloraminated water, and there is much concern regarding nitrosamines due to their potential carcinogenic and genotoxic effects, even at nanogram doses [20,21,22,23]. The US Environmental Protection Agency (EPA) has listed six N-nitrosamines in the Unregulated Contaminant Monitoring Rule 2 (UCMR2) to be monitored from 2008 to 2010 by public water systems [23]. The World Health Organization (WHO), Health Canada, and the Australian National Health and Medical Research Council have established a guideline value for N-nitrosodimethylamine (NDMA) in drinking water (100, 40 and 100 ng/L, respectively) [21,23]. The Ontario Ministry of the Environment and the California Department of Health Services set a more stringent notification level of 9 and 10 ng/L for NDMA, N-nitrosodiethylamine, and N-nitrosodiprophylamine. Similar permissible levels have emerged in some European Union (EU) member states, even if N-nitrosamines are not specifically listed in the EU Drinking Water Directive [21].

The aim of our study was to evaluate the possible formation of N-nitrosamines and other DBPs in hospital hot water networks treated with monochloramine. The effectiveness of this biocide in controlling *Legionella* spp. contamination was also verified. For this purpose, four different monochloramine-treated networks, in terms of the duration of treatment and method of biocide injection were investigated. Samples of untreated hot water, municipal cold water and, limited to the N-nitrosamines analysis, hot water treated with chlorine dioxide were analyzed for comparison.

## 2. Material and Methods

### 2.1. Hospital Setting

**Figure 1:**
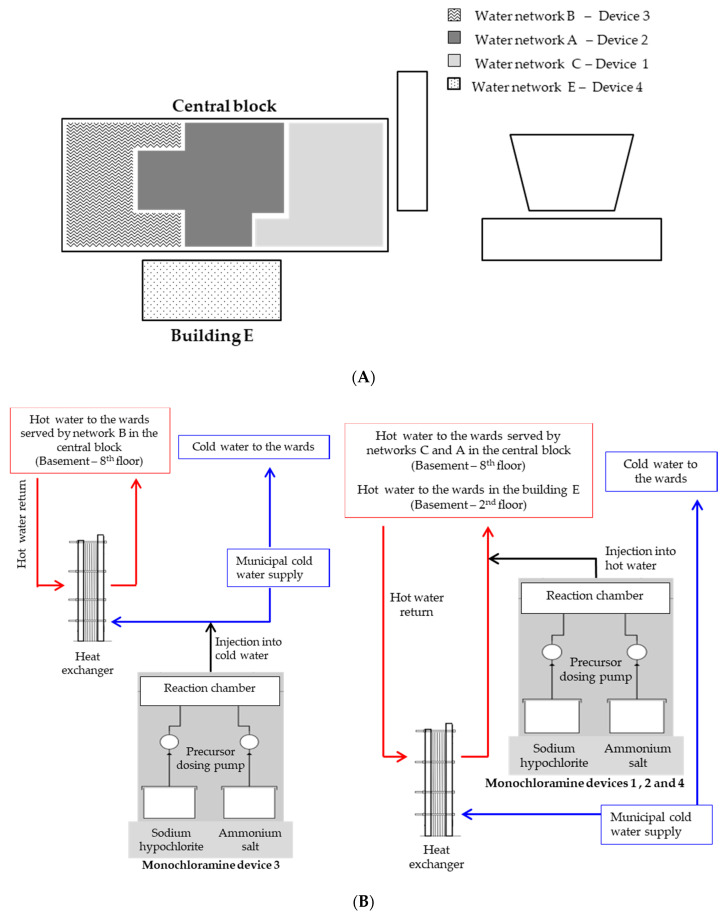
Schematic of hospital buildings (**A**) and monochloramine devices (**B**). White boxes in part A represent hospital buildings without monochloramine devices.

The study was conducted at the University Hospital Policlinico of Modena (Italy), a public 621-bed hospital consisting of a central nine-storey block and four separate buildings built between the 1970s and the 1990s. The same municipal water pipeline delivers water to all of the hospital buildings. A single main cold-water line branches out into several supply lines, each of which enters the water station of each hospital building. Once inside, cold water is heated using heat exchangers in order to produce hot water, which is moved in a water recirculating system. Three different water networks (A, B, and C) distribute hot water in parallel in the central block, while the other four buildings of the hospital have their own hot water pipelines (Figure 1A). Three monochloramine generators (Sanipur s.r.l., Brescia, Italy) are installed in the central block: Device 1 has been working on water pipeline C since March 2009 while, in July 2015, Devices 2 and 3 were installed on pipelines A and B—which had both been previously treated with chlorine dioxide since 2005. A fourth monochloramine generator (Sanipur s.r.l., Brescia, Italy) has been in operation since July 2012, in Building E (four floors, 20 years old), which is mainly devoted to operating theatre activity. Monochloramine is produced in situ from the chemical reaction between stabilized sodium hypochlorite and an ammonium salt, and is then continuously injected into the hot-water return (Device 1—loop C, Device 2—loop A, and Device 4—loop E), or into the cold make-up water pipeline (Device 3—loop B), as described in Figure 1B. The target concentration of monochloramine was between 2 and 4 mg/L at distal points, in accordance with the US EPA guidelines, which established a maximum monochloramine concentration of 4 mg/L [24].

### 2.2. Sample Collection

Over a 5 year period (January 2015–February 2020), 550 hot water samples (1 L) treated with monochloramine were collected from return loops and showers/taps for microbiological analysis, according to the hospital’s *Legionella* sampling and management plan. The protocol scheduled sampling at, at least, one remote point every 50 beds, reiterating (every 3 or 6 months) the same sites (sentinel taps), and testing other outlets based on the *Legionella* risk assessment. Samples were collected, without flaming and after flushing for 1 min, in sterile glass bottles containing sodium thiosulfate, in order to neutralize any residual disinfectant. At sampling, for each sample collected for *Legionella* testing, the water temperature (using a digital thermometer), pH (using a portable pHmeter), and monochloramine concentrations (using the Hach Lange indophenol method (Hach Lange, Milan, Italy); limit of detection (LOD) = 0.04 mg/L as combined chlorine) were measured.

Other chemical parameters such as chlorite, chlorate, total THMs, total organic carbon (TOC), nitrites, nitrates, and ammonia (NH_4_^+^) were measured on a more limited number of samples (about 20 samples/year) following the *Legionella* sampling program, with an increase in their monitoring for research purposes in the last period of the study, for a total of 136 water samples. The first sampling was performed when the chlorine dioxide devices were still operating in the A and B pipelines, and monochloramine had been working in pipelines C and E since 2009 and 2012, respectively. During each sampling session, a one liter sample was collected from the hot-water return loop of the four monochloramine-treated and one untreated networks, together with the cold water inlet to the hospital.

N-nitrosamines were measured on water samples collected during the three sampling sessions specifically organized for this study (December 2019, and January and February 2020); water samples were taken from the hot-water return loop and the two distal taps of the four monochloramine-treated networks, for a total of 36 one liter samples (12 samples for session); simultaneously, a total of 24 one liter control samples (8 samples for the session) were collected from the hot-water return circuit and two distal taps of an untreated and a chlorine dioxide-treated network, together with samples of cold water taken from the pipeline at the entrance to the hospital central block and to the Building E.

Acid-preserved glass bottles were used for chemical determinations.

All water samples were returned to the laboratory immediately after collection and analyzed within 24 h.

### 2.3. Laboratory Methods

#### 2.3.1. Microbiological Analysis

Legionellae were isolated by a culture method in accordance with the ISO 11731:1998, as previously reported [15]. Briefly, 1 L of water was filtered (using a 0.2-μm-pore-size polyamide filter, Millipore, Billerica, MA, USA), the filtrate was suspended in 10 mL of the original sample water by vortexing for 2 min, and 5 mL of the sample heat-treated. Two aliquots of 200 μL of the original and concentrated samples (heat-treated and untreated) were plated onto a GVPC selective medium (Thermo Fisher Scientific, Waltham, MA, USA). The plates were incubated at 36 ± 1 °C with 2.5% CO_2_ for 10 days and analyzed on Day 4 with a dissecting microscope. Presumptive *Legionella* colonies were subcultured on BCYE (with cysteine) and CYE (cysteine-free) media (Thermo Fisher Scientific) and then incubated at 36 ± 1 °C for 48 h. Colonies grown on BCYE were subsequently identified to the species and serogroup levels using an agglutination test (Thermo Scientific™ Legionella Latex Test). The results were expressed as the number of colony forming units (cfu) per liter, and the LOD of the procedure was 25 cfu/L. Only viable planktonic bacteria were enumerated.

#### 2.3.2. Chemical Analysis

Chlorites and chlorates were analyzed using ion chromatography (the EPA method 300.1, LOD = 50 and 60 µg/L for chlorites and chlorates, respectively), THMs (chloroform, bromodichloromethane, dibromochloromethane and bromoform) by gas chromatography (the modified EPA method 551.1 [25], LOD for each THM sample = 0.01 μg/L), TOC by infrared spectroscopy (the APAT CNR-IRSA method 5040, LOD = 0.1 mg/L), nitrites, nitrates, and free ammonia using specific colorimetric methods (Hach Lange, Milan, Italy). In detail, we used the diazotization method for nitrites (method 10019, LOD = 0.001 mg/L), the chromotropic acid method for nitrates (method 10020, LOD = 0.2 mg/L), the indophenol method for ammonia in the chloraminated samples (method 10200, LOD = 0.02 mg/L) and the salicylate method for ammonia in the monochloramine-free water (method 10023, LOD = 0.02 mg/L).

Nine N-nitrosamines (N-nitrosodipropylamine (NDPA), N-nitrosodibutilamine (NDBA), N-nitrosodiethylamine (NDEA), N-nitrosodimethylamine (NDMA), N-nitrosomorpholine (NMOR), N-nitrosopiperidine (NPIP), N-nitrosopyrrolidine (NPYR), N-nitrosodiphenylamine (NDPHA), and N-nitrosomethylethylamine (NMEA)) extracted with dichloromethane were measured using gas chromatography coupled to a mass spectrometer (EPA SW-846 Methods 3510C [26]). The LODs for each N-nitrosamine were: 2.3 ng/L for NPIP, 2.1 ng/L for NDEA, 2.0 ng/L for NDBA and NDMA, 1.8 ng/L for NDPA and NDPHA, 1.7 ng/L for NPYR, 1.0 ng/L for NMOR, and 0.9 ng/L for NMEA.

Italian and European legislations set the parametric values for drinking water at 700 μg/L for chlorite, 30 μg/L for the total THMs, 0.50 mg/L for nitrite and ammonium, and 50 mg/L for nitrate [27,28]. In Italy and in the EU, N-nitrosamines and chlorate are not listed in the Drinking Water Directive. Provisional standard values were proposed in the Netherlands (12 ng/L for NDMA), and in Germany (10 ng/L for NDMA and NMOR) [21]. The WHO established a provisional value of 700 µg/L for chlorate and a guideline value of 100 ng/L for NDMA in drinking water [29].

#### 2.3.3. Corrective Actions

Corrective actions were taken to ensure that the proportions of the chlorine and ammonia precursors during the monochloramine formation were correct. These corrective actions included the draining/cleaning of the hypochlorite storage tanks, the frequent replacement with fresh products, use of a more stabilized hypochlorite solution (diluted, filtered, and pH adjusted), and monochloramine set point dosage adjustment.

#### 2.3.4. Statistical Analysis

Statistical calculations were performed using PASW statistics version 25.0 (SPSS Inc, Chicago, IL, USA). Logarithmic transformations were used to normalize the bacteriological data, and the results are presented as geometric mean values. The chi-square test and one-way analysis of variance (ANOVA) with the Bonferroni test were applied, whenever necessary.

## 3. Results

Only 22 out of 550 samples (4.0%), collected over 5 years from the monochloramine-treated hot water networks, were culture-positive for *Legionella* spp. at low levels (geometric mean 1.9 × 10^2^ cfu/L), although most isolates (13/22, 59.1%) belonged to serogroup 1. Table 1 reports the numbers and percentages of *Legionella* positive samples and their geometric means, together with the mean values of monochloramine, temperature, and pH, according to the four treated pipelines. Water pipework significantly affected the percentage of positive samples (χ^2^ = 12.656, *p* < 0.005) but not their bacterial load (F = 2.294, ns). Network C showed the lowest percentage, followed by the A, B, and E pipeworks, but all of them, ranging from 0.8 to 9.1%, were largely below 30%—a value reported as being an indicator of low risk for disease transmission. No significant difference in monochloramine mean concentration was found between water networks, even if 25/136 samples (18.4%) in loop B had a monochloramine content >4 mg/L, compared to 22/172 (12.8%), 12/121 (9.9%), and 8/121 (6.6%) in the A, C and E loops, respectively. Network A had a mean temperature significantly higher than the other networks (*p* < 0.05 by the Bonferroni test), although still in a range (45–50 °C) that prevents the degradation of the biocide. A neutral pH was always found in all the treated pipelines.

N-nitrosamines were all below the LOD in monochloramine and chlorine dioxide-treated and untreated water, as well as in the cold water entering the hospital buildings.

Chlorites were only detected in July 2015, in the A and B loops, when the chlorine dioxide devices were still operating before the application of monochloramine (1764 and 1748 µg/L, respectively).

**Figure 2:**
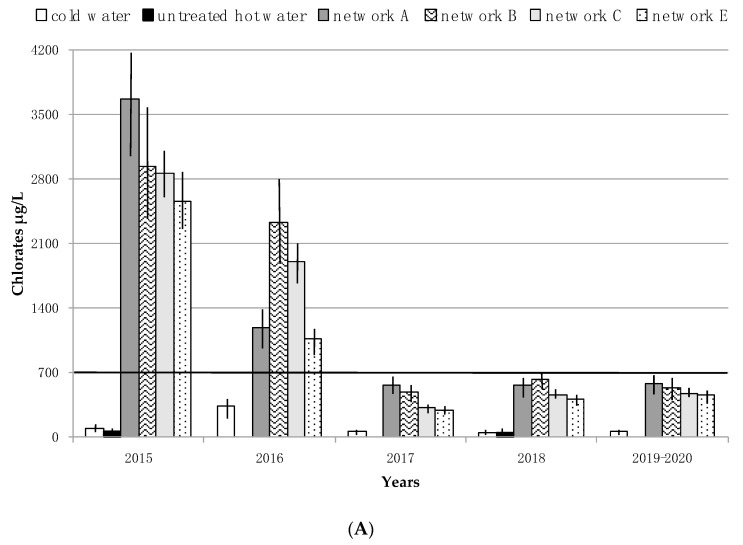

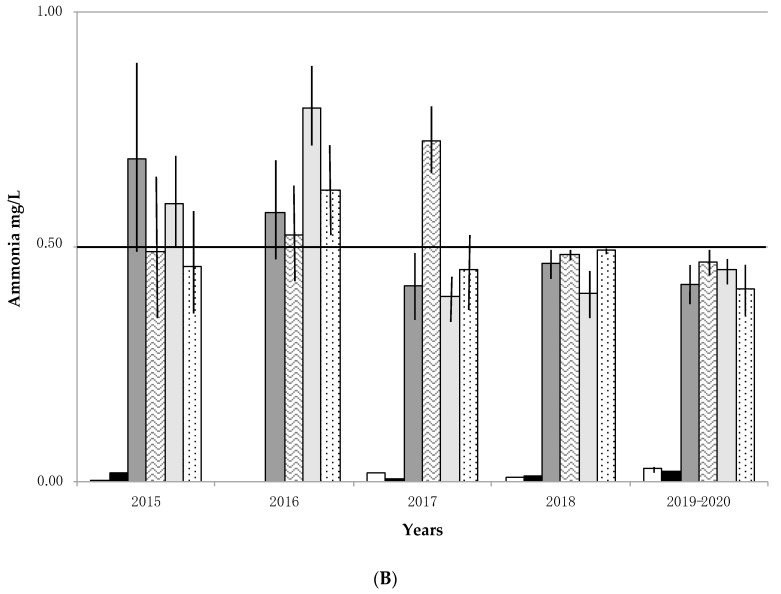
Trend of chlorates (**A**) and ammonia (**B**) expressed as mean ± Standard Deviation (SD) according to sampling sessions and water pipelines. Black line (700 μg/L) indicates the provisional value for chlorate in drinking water, established by WHO. Black line (0.50 mg/L) indicates the maximum permissible drinking water ammonium value established by Italian and European legislation.

Chlorates ranged between LOD (<60) and 352 µg/L in cold water, and between LOD (<60) and 240 µg/L in the untreated hot water loop. In the monochloramine-treated pipelines, chlorate concentrations were always higher than the WHO value of 700 µg/L during the first 2 years of the study, without any significant differences between the four devices (Figure 2A). Within the same time frame, the ammonia concentrations of the treated samples increased compared to untreated hot water and cold water (Figure 2B). In light of these results, corrective actions were regularly taken over the next 3 years, as previously described (see the chapter on *Corrective actions*). As a result, since 2017 the chlorates decreased below the WHO provisional value in all the water networks, and free ammonia below 0.50 mg/L, with the exception of pipeline B for the first months of 2017 (Figure 2A,B).

TOC concentrations were below the LOD in 63% of the samples, and at very low levels (0.1–0.3 mg/L) in the remaining. THMs were extremely low as they were <1 μg/L for all systems. In all the sample nitrites and nitrates were below the limits imposed by Italian and European regulations (range 0.001–0.072 mg/L and 4.30–22.00 mg/L, respectively), and they did not increase in the presence of monochloramine.

## 4. Discussion

Our findings confirm the long-term effectiveness of the monochloramine device operating since 2009 in controlling *Legionella* contamination, as reported in previous studies [15,17,30,31]. Similarly, the monochloramine devices installed at the beginning of this study, in two water networks previously treated with chlorine dioxide, proved to be effective. Indeed, approximately 30% (60 positives/201 samples) of the samples taken from these pipelines over the three-year period, before chlorine dioxide was replaced with monochloramine, tested positive for *Legionella* [17]. The same effectiveness was verified in Building E, where the percentage of contamination in the period 2001–June 2012, before the installation of the monochloramine device, was 89.9% (80/89 samples), falling to 11.6% (21/181) over the eight years following its placement. The monochloramine effectiveness is also confirmed, irrespective of biocide injection point. However, injecting monochloramine into the cold make-up water could lead to a more difficult control of biocide concentration, as highlighted by the higher percentage of samples exceeding the maximum contaminant level established by the EPA in loop B. In light of this observation, it could be advisable to dose monochloramine directly in the hot water loop. Three chloramines are produced when chlorine reacts with ammonia in water: monochloramine, dichloramine, and trichloramine. Monochloramine is generally the predominant species at neutral pH and above [32,33]. As pH plays a key role in the formation of the monochloramine from precursors, recurring pH values above 7.0 prove that monochloramine is the predominant species in our system, without any formation of toxic and odorous di- and tri-chloramines.

To our knowledge, this is the first study that includes nitrosamines in the assessment of the formation of potentially dangerous DBPs during long-term monochloramine treatment in small-scale plants. In our hospital, no formation of the most prevalent N-nitrosamines was observed, even in the hot-water treated with monochloramine for nine years.

The low levels of nitrosamine precursors such as TOC, nitrites, and nitrates found in the hospital tap water may explain this satisfactory result. Indeed, it is known that N-nitrosamine formation during water treatment requires the presence of organic nitrogen-containing precursors [20,21,22,34]. The presence of nitrite in a water supply is undesirable because of health concerns (e.g., methaemoglobinaemia in infants) [29]. In our study, no increase in nitrite levels occurred by using monochloramine, thus supporting the safety of this biocide.

The measured THMs concentrations were extremely low in all water networks, which was in line with the results of our previous study where, in addition to THMs, haloacetic acids were also investigated, and were not detected in the network treated with monochloramine [15]. These findings confirm in a small-scale hot-water system what has already been observed in drinking water, namely that using monochloramine instead of free chlorine as a residual disinfectant minimizes the formation of these regulated toxic DBPs [13,14].

Congenital anomalies, the impairment of neurobehavioral and neurological development, the delay in female sexual development, soft tissue anomalies, and altered thyroid function were observed in association with chlorate exposure via drinking water [35]. Chlorate formation in chloraminated water is due to hypochlorite degradation when it is improperly stored and/or of poor quality [29]. Therefore, we suggest using a stable formulation of hypochlorite, and replacing this precursor frequently with a fresh product, in order to avoid the incomplete reaction of monochloramine precursors and the unintended build-up of chlorates and free ammonia. Routine monitoring of free ammonia is also recommended because ammonia can compromise disinfection efficiency, can lead to nitrite formation in distribution systems, and can cause the failure of manganese removal filters, as well as taste and odor problems. Other authors have observed nitrification in water due to a non-stoichiometric dosage of the monochloramine precursors, and the re-growth of denitrifying bacteria following long-term treatment in hospital [36]. However, the limited increase in free ammonia, above the EU drinking water standard of 0.50 mg/L, is not relevant to public health. Indeed, ammonia in water is an indicator of possible bacterial, sewage, and animal waste pollution, and exposure from environmental sources is considered insignificant in comparison with the endogenous synthesis of ammonia [29].

We emphasize that monochloramine does not produce chlorites, which are regulated at national and international level due to their toxicity [35]. This is another aspect supporting the use of monochloramine as an alternative to chlorine dioxide.

A limitation of this study is the lack of information on the potential presence of other DBPs, such as haloketones, chloropicrin, aldehydes, etc., previously found in water treated with monochloramine [37], but not currently regulated by the EU Drinking Water Directive. Another limitation is its monocentric nature. The single-hospital setting may limit the generalizability of the results. Further research is required for dealing with these issues.

## 5. Conclusions

National and international guidelines recommend controlling *Legionella* spp. contamination, with particular reference to healthcare structures. Disinfection procedures are required to prevent or control the risk of *Legionella* water contamination, and each procedure has different advantages, as well as maybe posing a risk to human health.

Thanks to our decades of experience in the use of monochloramine for the control of *Legionella* spp. contamination in different water systems of the same hospital, we can affirm that this biocide, if properly produced, continuously dosed, and regularly maintained is extremely effective against *Legionella* and does not produce any toxic disinfection by-products, such as nitrosamines and chlorites, or nitrification.

In conclusion, our study confirms that monochloramine may be proposed as an effective and safe strategy for the continuous disinfection of building plumbing systems, preventing vulnerable individuals from being exposed to legionellae and to dangerous DBPs.

## Figures and Tables

**Table 1:** Microbiological and physical–chemical parameters in water samples collected from the four monochloramine-treated networks.

Water Pipework	Samples Number	*Legionella* spp. Positives		Physical-Chemical Parameters		
		*n*/Total (%) ^a^	Geometric Mean cfu/L (Range)	Monochloramine Mean ± SD mg/L	T (°C) Mean ± SD	pH Mean ± SD
A	172	4/172 (2.3)	1.3 × 10^2^ (25–2.9 × 10^3^)	2.84 ±1.18	45.3 ± 4.5 ^b^	7.4 ± 0.3
B	136	6/136 (4.4)	7.2 × 10 (25–3.0 × 10^2^)	2.98 ± 1.10	39.1 ± 6.6	7.5 ± 0.4
C	121	1/121 (0.8)	2.5 × 10^3^	2.82 ± 0.95	40.9 ± 5.0	7.4 ± 0.2
E	121	11/121 (9.1)	2.9 × 10^2^ (25–3.2 × 10^3^)	2.93 ± 0.90	40.7 ± 8.1	7.5 ± 0.2
Total	550	22/550 (4.0)	1.9 × 10^2^ (25–3.2 × 10^3^)	2.89 ± 1.05	41.8 ± 6.6	7.4 ± 0.3

SD = Standard Deviation ^a^ *p* < 0.005 ^b^ *p* < 0.05 A vs. all the other networks.
